# Under Fire: High Survival Rates of a Critically Endangered Marsupial During a Low‐Severity Prescribed Burn

**DOI:** 10.1002/ece3.74637

**Published:** 2026-11-05

**Authors:** Harry Moore, Shelby Middleton, Dale Nimmo, Ciara McIlduff, Nicole Willers

**Affiliations:** ^1^ Department of Biodiversity, Conservation and Attractions Perth Western Australia Australia; ^2^ University of Western Australia Perth Western Australia Australia; ^3^ Gulbali Institute, Charles Sturt University Albury New South Wales Australia

**Keywords:** biologging, climate change, conservation management, fire, fire severity, hollow‐bearing trees, prescribed burning, threatened species

## Abstract

Climate change is intensifying fire weather globally, increasing land managers' reliance on prescribed burning to reduce fuel loads and mitigate wildfire risk. Yet the direct effects of prescribed fire on threatened wildlife remain poorly understood, partly because individual‐level data on survival and behaviour during fire events are exceptionally rare. We used VHF telemetry and accelerometry to track the survival, space use and diel activity of nine western ringtail possums (*Pseudocheirus occidentalis*; ngwayir; eight collared, one uncollared joey)—a Critically Endangered arboreal marsupial widely regarded as highly sensitive to fire—through a low‐ to moderate‐severity prescribed burn at Yalgorup National Park, south‐west Western Australia. All nine animals survived direct fire exposure despite several refuge trees being scorched or structurally damaged. Post‐fire, possums relocated an average of 181 m, but movement distances were unrelated to burn severity and possums showed no selection for unburnt habitat, suggesting limited spatial disruption at the home‐range scale. This may partly reflect the predominantly low‐severity nature of the burn (95.7% of the burnt area). Accelerometry revealed suppressed nocturnal activity during smoke exposure and the fire period, with only partial recovery in the days following the burn, indicating measurable sublethal costs even where direct mortality did not occur. We interpret these behavioural responses as potentially consistent with adaptations shaped by long‐term exposure to frequent, low‐severity fire regimes historically associated with Noongar burning on the Swan Coastal Plain. Although based on a small number of individuals at a single site, and a carefully implemented low‐ to moderate‐severity prescribed burn, our findings provide rare empirical evidence that a critically endangered marsupial can survive prescribed fire. These results have implications for how prescribed burning programs are designed and implemented in the context of evolving fire regimes under climate change.

## Graphical abstract

Using telemetry and accelerometry, we tracked nine critically endangered western ringtail possums (*Pseudocheirus occidentalis*) through a low‐ to moderate‐severity prescribed burn at Yalgorup National Park, south‐west Western Australia, and found that all individuals survived direct fire exposure. While activity levels declined during and immediately after the burn, possums showed minimal changes in movement patterns, habitat selection and space use, indicating resilience to low‐severity fire and providing rare empirical evidence of survival and behavioural responses to prescribed fire in this critically endangered marsupial.

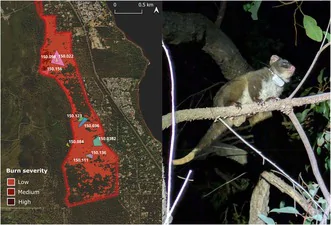

## 1 Introduction

Across much of the world, shifting fire regimes are emerging as an escalating management challenge (Cunningham et al. 2024). Climate change is projected to intensify extreme fire weather, leading to more frequent, severe and widespread wildfire conditions with longer fire seasons (IPCC 2022). In response, land managers are increasingly utilising prescribed burning programs to reduce accumulated fuel loads and mitigate the risk of catastrophic wildfire to communities, infrastructure and biodiversity assets. Doing so responsibly requires a robust understanding of the potential impacts of burning on biodiversity values, including threatened species—yet this understanding is often absent (Gordon et al. 2025; Doherty et al. 2026).

Despite the ubiquity of fire as a management tool, direct mortality of animals during fire events remains poorly understood (Jolly et al. 2022). Anecdotal accounts of mass mortality are well documented—most dramatically during the 2019–2020 Australian megafires (Driscoll et al. 2024), estimated to have killed or displaced more than three billion animals (van Eeden and Dickman 2023)—yet rigorous, individual‐level data on survival during fire remain exceptionally rare. This is largely a logistical problem: fires are difficult to anticipate at the scale of individual animals, pre‐fire baselines are rarely in place, and the window between ignition and impact is often too narrow for targeted monitoring (Jolly et al. 2022). Where individual‐level data do exist, they derive almost exclusively from telemetry studies conducted around prescribed burns (e.g., Povh et al. 2023), and are biased towards open or semi‐open habitats where tracking is logistically feasible (e.g., Fenner and Bull 2007). Few studies have focused on arboreal mammals, and none to our knowledge have tracked critically endangered species through fire events. The absence of empirical data documenting the effects of fire, particularly anthropogenic burns, has also contributed to confusion and unsupported assertions about fauna fire responses (Doherty 2026).

The western ringtail possum (*Pseudocheirus occidentalis*), known as ngwayir by Noongar people on whose Country this study occurs, is emblematic of the conservation challenge this knowledge gap creates. Listed as Critically Endangered under both the Australian EPBC Act (DCCEEW 2026) and the IUCN Red List (Burbidge and Zichy‐Woinarski 2017), the species has declined substantially since European settlement and is now largely restricted to coastal woodland in south‐west Western Australia (Teale et al. 2025). A nocturnal folivore strongly associated with peppermint (*Agonis flexuosa*) woodland across much of its range, the ngwayir shelters during the day in tree hollows as well as self‐constructed dreys (Baker and Gynther 2023). Major threats to ngwayir include habitat loss and fragmentation (Wayne et al. 2006, 2), predation by introduced species such as feral cats and red foxes, and the increasing frequency of heatwaves and drought conditions driven by climate change (Department of Parks and Wildlife 2017; Moore et al. 2026). In addition, fire, including prescribed burning, has also been identified as a high impact threat to ngwayir (Doherty et al. 2026).

Previous attempts to quantify the impact of fire on ngwayir have largely focused on changes to key habitat features as well as population‐scale effects. For example, Inions et al. (1989) found that a high‐severity prescribed burn destroyed 38% of previously used den trees, substantially reducing hollow availability in the short term. At the population level, Wayne et al. (2022) reported that the incidence of severe fire in the past 20 years was the strongest predictor of ngwayir abundance in the southern jarrah forest, and Wayne et al. (2006) similarly found higher ngwayir presence at sites that were long unburnt or burnt at low severity. More recently, Wayne et al. (2026) incorporated prescribed‐burn history into long‐term spotlight‐monitoring analyses and found that fire age had no measurable effect on ngwayir detection probability or inferred abundance. One study has attempted to document individual‐level fire impacts, reporting that 17 of 22 ngwayir died during a prescribed burn in tuart–banksia woodland; however, this study did not track the fate of individual animals but instead estimated mortality based on an unreported number of carcasses located beneath hollows, indirect signs of mortality (e.g., flies near previously occupied hollows), and by assuming that individuals that were not re‐sighted after the fire had died (Zylstra 2023). As such, it remains unclear which mechanisms pose the greatest threat to ngwayir during fire—whether direct exposure to heat, asphyxiation from smoke or the destruction of key shelter sites. Moreover, it remains unclear whether ngwayir exhibit behavioural traits shaped by long evolutionary exposure to frequent, low‐severity Noongar burning that could potentially mitigate these impacts, or whether contemporary fire regimes now exceed the conditions to which their fire‐response behaviours are adapted. This distinction has important implications for assessing sensitivity to fire.

Here we address this gap directly, using individual‐level VHF telemetry and accelerometry to monitor the survival, space use, and activity of ngwayir through a prescribed burn at Yalgorup National Park, Western Australia. We specifically aimed to (i) determine short‐term survival outcomes following direct fire exposure, (ii) assess whether fire altered post‐fire movement distances and habitat selection and (iii) characterise changes in diel activity patterns across the smoke‐exposure, fire and post‐fire periods, with a view to evaluating whether observed responses are consistent with fire‐adapted or fire‐naive behaviour. To our knowledge, this is the first study to track individual ngwayir through a prescribed burn, and one of very few to document immediate individual‐level responses of any hollow‐dependent marsupial to fire.

## 2 Methods

### 2.1 Study Area

This study took place within a 235‐ha area of the northern section of Yalgorup National Park, approximately 100 km south of Perth on the Swan Coastal Plain, Western Australia (Figure 1). This area is the most northern section of the ngwayir's contemporary geographic range (Teale et al. 2025). Vegetation within the park is characterised by a mosaic of tuart ( *Eucalyptus gomphocephala* ), marri ( *Corymbia calophylla* ), jarrah ( *Eucalyptus marginata* ) and peppermint (*Agonis flexuosa*) woodland with an understorey of bull banksia (*Banksia grandis*), sheoak (*Allocasuarina fraseriana*), grass trees (*Xanthorrhoea* spp.), zamia ( *Macrozamia riedlei* ; Archibald et al. 2005; Figures S1–S3). The region experiences a Mediterranean climate, with warm dry summers and mild wet winters. Mean annual rainfall is approximately 605 mm, strongly winter‐dominated, with the wettest months (June and July) each receiving around 119–127 mm and the driest months (December and February) receiving as little as 12–14 mm on average (Bureau of Meteorology 2026). Mean maximum temperatures range from 30°C in February to 18°C in July, while overnight minima span from around 11°C in July to 19°C in February. Within the study polygon (235 ha), the majority of the area (59.4%, 139.51 ha) was last burnt in 2015, followed by 33.4% (78.55 ha) last burnt in 2004, and 5.9% (13.83 ha) last burnt in 2003. A small proportion of the polygon (1.1%, 2.70 ha) had no recorded fire history. Over the 30‐year recording period (1985–2015), the summed area burnt across all recorded fire events was 875.43 ha (including areas that burnt more than once; DBCA 2026).

**FIGURE 1:**
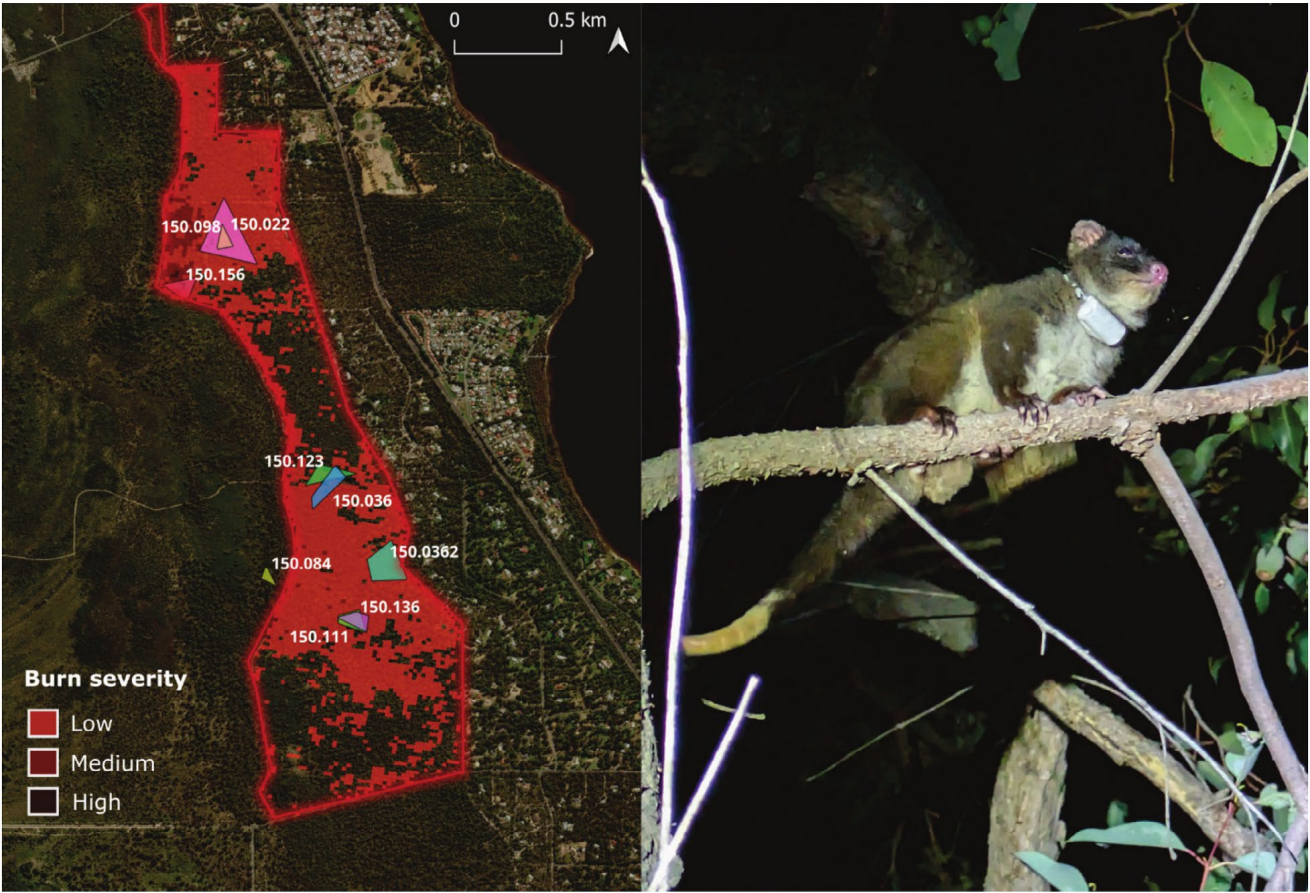
Study area and focal species. (Left) Satellite imagery of the SWC_118 prescribed burn cell at Yalgorup National Park, showing the fire scar (red) and minimum convex polygon home ranges derived from VHF telemetry locations of collared western ringtail possums (*Pseudocheirus occidentalis*) or ngwayir. (Right) A collared western ringtail possum, fitted with an Axy‐5 VHF accelerometer collar during the study.

### 2.2 Study Species

Ngwayir are nocturnal folivores, strongly associated with peppermint (*Agonis flexuosa*) forest and woodland, although they also occupy jarrah, wandoo, marri and mixed myrtaceous vegetation (Jones et al. 1994). Breeding occurs in late autumn to winter, with litters of one to three emerging from the pouch at around 3 months of age (Jones et al. 1994). Ngwayir lifespans exceeding 6 years have been recorded in the wild (Baker and Gynther 2023). The primary threats to the species are habitat loss and fragmentation associated with urban expansion, predation by introduced red foxes and feral cats, inappropriate fire regimes, and the projected effects of a warming and drying climate on both ngwayir and the vegetation communities on which they depend (Department of Parks and Wildlife 2017). Ngwayir are particularly vulnerable to dehydration under high temperatures (Moore et al. 2026), and to predation where canopy cover has been disrupted (Burbidge and Zichy‐Woinarski 2017).

Three months prior to the prescribed burn, the study area was systematically surveyed for ngwayir and common brushtail possums ( *Trichosurus vulpecula* ) using spotlighting transects spaced 100 m apart across the 235‐ha burn cell. In total, 25,705 m of transects were surveyed. These surveys confirmed active use of the area by both species, with 22 western ringtail possums and 17 brushtail possums detected.

### 2.3 Collaring

Possums were captured using the pole‐hook capture method (described in Turner 2022). Once captured, possums were placed in cloth bags with their eyes covered to minimise stress. All animals were weighed and sexed, and only those > 700 g and without pouch young were collared. All animals were released at their point of capture immediately after processing. At the end of the study period, all collared possums were recaptured using the same methods. Collars weighed ~22.5 g (< 3.5% of a 700 g possum's body mass) and incorporated a cotton weak‐link to ensure detachment if animals were not recaptured.

A total of nine possums (F = 5, M = 4) were collared in the week preceding the burn. Six individuals were collared on 29 April, two on 30 April, and one on 2 May. All animals were captured within the boundary of the proposed burn cell. Each possum was physically assessed and deemed to be in good condition, and no individuals were carrying pouch young. One female was captured with a 650 g joey; however, the joey was below the 700 g collaring limit and therefore was not fitted with a collar. However, because the joey remained in the immediate vicinity of its mother throughout the study and therefore stayed readily trackable, its fate post‐fire could be determined reliably.

Possums were fitted with Axy‐5 VHF accelerometer collars (TechnoSmart 2026). Accelerometers recorded tri‐axial acceleration at 10 Hz (±4 g) across surge (forward–backward), heave (up–down) and sway (side‐to‐side) axes. Vectorial Dynamic Body Acceleration (VeDBA), a widely used proxy for activity and energy expenditure (Qasem et al. 2012), was calculated as:

(1) $$\text{VeDBA}=\sqrt{A_{x}^{2}+A_{y}^{2}+}A_{z}^{2}$$

where $A_{x}$, $A_{y}$, $A_{z}$ and are dynamic body acceleration values for each axis. VeDBA values were averaged into 1‐min intervals for each individual to generate fine‐scale diel activity profiles.

Possums were VHF‐tracked to their daytime refuge on 30 April, 1 May, 2 May, 3 May, 4 May, 5 May, 6 May, 7 May and 11 May 2026. Refuge attributes were recorded for each den tree, including tree species, diameter at breast height (DBH), tree height, refuge height, and whether the refuge entrance was visible from the ground. Where entrances were visible, we also visually estimated hollow characteristics, including entrance dimensions and hollow wall thickness. Both tree height and refuge height were measured using a laser rangefinder.

Tracking on 4 May and 5 May coincided with the first and second days of the prescribed burn, respectively; on both days, refuge locations and attributes were recorded prior to ignition to ensure animals were located safely before fire operations commenced. Possums were also tracked nocturnally on 7 May, after the burn had occurred. Nocturnal tracking allowed us to visually confirm possum post‐fire condition and verify that collared animals were uninjured/alive. A final set of refuge locations was recorded on 11 May. All capture, handling and monitoring procedures were carried out under the Western Australian Department of Biodiversity, Conservation and Attractions (DBCA) Animal Ethics Committee approval 2026‐04A and a Section 40 Authorisation to take or disturb threatened species (TFA‐2425‐0063).

### 2.4 Prescribed Burn

The prescribed burn at Yalgorup National Park encompassed a 235.1‐ha target cell and was conducted on 4–5 May 2026. Prior to the burn, five vegetation transect surveys were conducted throughout the cell. Fuel loads (measured as the amount of burnable material) were estimated using the Forest Fire Behaviour Tables (Sneeuwjagt and Peet 1998) field assessment protocol. Each transect comprised a 100 m line positioned in fuels representative of the target vegetation type, beginning at least 150–200 m from the burn boundary to avoid edge effects (DBCA and DFES 2020). At each of 10 sample points spaced 10 m apart, litter depth was measured using a litter depth gauge, trash (dead twigs, bracken and woody debris ≥ 10 mm diameter) and scrub components were visually assessed within a 3 m radius of each point (DBCA and DFES 2020). Average values per transect were converted to fuel weights (t/ha) using FFBT lookup tables and summed across litter, trash and scrub components to give a total available fuel estimate. These surveys suggested that fuel loads ranged from 10.8 to 23.2 t/ha.

Pre‐fire preparation for this burn was expanded beyond normal DBCA practice, reflecting the high number of western ringtail possums in the burn area. Under standard conditions, DBCA staff ‘treat’ habitat trees—large mature trees typically > 1 m DBH with well‐developed hollows or structural features that provide shelter or nesting sites for fauna—by removing surface fuels such as leaf litter, bark and fine debris from around their bases using raking and blowing. This is done to reduce the likelihood of the tree being consumed during the burn, or compromised to the extent that it no longer provides suitable refuge for fauna. This treatment is typically applied only within the first ~50–100 m from the burn edge for burn security; however, for this burn crews extended fuel‐removal work well into the core of the reserve. This expanded fuel‐removal work is consistent with the species‐specific guidance in the ‘Ngwayir (Western Ringtail Possum) Fire Management Guideline’ (Wayne 2006), which emphasises protecting hollow‐bearing trees and other key shelter structures during prescribed burns to minimise impacts on possums. To avoid biasing the study, staff were not informed of the specific locations of hollows or possum refuge sites.

The success criteria for the burn required 70%–90% of the cell to be burnt, with no more than 40% occurring at high or very high severity. Ignition commenced at approximately 10:00 on 4 May 2026 under observed conditions of 14.8°C and 71% relative humidity at 09:00, with light ESE winds (6 km/h), rising to a maximum temperature of 20.9°C later in the day. Winds shifted to WSW during the afternoon, with a maximum gust of 26 km/h recorded at 15:05 (Bureau of Meteorology 2026). Approximately 40% of the cell was treated on the first day. On 5 May 2026, ignition of the remaining 60% proceeded through the morning as a surface inversion lifted around 1000–1100 WST. Observed conditions at 09:00 were 15.1°C, 70% relative humidity and light easterly winds (4 km/h), with a maximum temperature of 20.5°C reached later in the day. Winds again shifted to WSW during the afternoon, with a maximum gust of 24 km/h recorded at 14:48.

The burn was implemented with lighting techniques to promote low‐severity fire behaviour (Figures S4–S7). Fine‐scale spot ignition was applied at generally 40–80 m spacing and adjusted to conditions, with lighting placed downwind of hollow‐bearing trees to encourage backing fire and prevent canopy scorch. Crews wetted down key habitat trees and logs where required, and ignition was applied conservatively, with grass trees only ignited where necessary for burn security. The extent of these mitigation measures was not quantified and therefore could not be incorporated into subsequent analyses. Fire spread remained slow and patchy across the cell, typically advancing at 18–30 m/h with low flame heights observed (0.1–0.5 m), and efforts were made to minimise machinery use and vehicle disturbance throughout the burn area.

Following the burn, Sentinel‐2 satellite imagery was used to map burn extent and severity following DBCA's standard fire severity mapping approach (van Dongen et al. 2023). Burn severity was assessed using a differenced Normalised Burn Ratio (dNBR) methodology, whereby near‐infrared and shortwave‐infrared reflectance measured before and after the fire were compared to quantify fire‐induced changes in vegetation. Areas were subsequently classified as unburnt, low, moderate or high severity according to the magnitude of change detected and coded numerically from 1 (unburnt) to 4 (high severity) for use as a continuous covariate in subsequent analyses.

A total area of 136.3 ha was burnt across 4–5 May, representing 58.6% of the burn cell (See Supplementary Material). While the majority of the burnt area experienced low‐severity fire (130.4 ha, 95.7% of the burnt area), moderate‐ to high‐severity burning occurred in the northern section of the cell (5.9 ha, 4.4% of the burnt area—5.5 ha moderate, 0.4 ha high) where dense stands of grass trees, which are extremely flammable, produced more intense fire behaviour. The southernmost section of the cell, dominated by thicker stands of tuart and peppermint, achieved less ignition and fuel consumption due to the denser, less combustible structure of the vegetation. Across most of the cell, canopy cover remained largely intact, consistent with the low‐severity nature of the burn.

To assess the accuracy of the mapped burn scar, we evaluated 50 randomly selected points across the burn cell. Of these, 49 (98%) matched the mapped fire boundary, while one point indicated evidence of burning despite being located 40 m beyond the nearest mapped burn edge. This high level of agreement suggests that the satellite‐derived burn scar provided a reliable representation of the fire extent.

### 2.5 Refuges

To estimate the impact of the burn on possum refuges, all refuge trees known to be used by possums prior to ignition were visually assessed following the burn. For each refuge tree, we recorded scorch height, the proximity of burn evidence and any structural damage such as limb loss or hollow degradation. These assessments allowed us to quantify how fire affected the condition and safety of previously used refuges.

### 2.6 Statistical Analysis

#### 2.6.1 Possum Activity

All statistical analyses were performed in R version 4.4.1 (R Core Team 2024). To examine whether the diel activity pattern of ngwayir differed among fire‐related periods, we fitted a generalised additive model (GAM) using the bam function in the mgcv package (Wood 2000), with the log of one‐minute mean VeDBA as the response variable, modelled with a Gaussian distribution and AR(1) errors to account for autocorrelation between consecutive 1‐min observations. Each possum's accelerometer data were assigned to one of four periods relative to the time of ignition at their refuge location: baseline (< −24 h), smoke exposure (−24 to 0 h), fire (0 to +24 h) and post‐fire (> +24 h). The smoke‐exposure period was assigned to Day 2 possums during the 24 h before ignition reached their refuge locations, when fire was already active elsewhere within the burn cell. One individual (150.084) was unexposed to fire and contributed data only to the baseline and smoke‐exposure periods. Time of day was represented as a circular penalised regression spline (bs = ‘cc’, k = 15, knots at 0 and 24 h), with separate smooths estimated for each period via a period‐by‐smooth interaction. Fire period and sex were included as parametric fixed effects, with baseline as the reference level, because they are the factors whose effects we tested. Individual was included as a random intercept (a random‐effect smooth, bs = ‘re’) because the possums are a sample from a wider population and were measured repeatedly; this accounts for repeated measures and for differences in baseline activity between individuals. Support for period was assessed using AIC (maximum likelihood) by comparing the model with a single diel curve shared across periods and no period effect.

To compare possum activity among fire‐related periods during the day and night separately, we fitted two linear mixed models (LMMs) with a Gaussian distribution, implemented in the lme4 package (Bates et al. 2015). The response variable was the log of the mean 1‐min VeDBA within each diurnal or nocturnal window, calculated separately for each possum on each day. Data were split into daytime (06:00–18:00) and night‐time (18:00–06:00) windows. Each model included period as a fixed effect (baseline, smoke exposure, fire, post‐fire), with individual identity as a random intercept to account for repeated measures. Period assignment and the treatment of the unexposed individual (150.084) followed the approach described above.

To test whether burn severity influenced the size of the activity response to fire, we fitted two further linear mixed models (diurnal and nocturnal), using each possum's deviation in mean log(VeDBA) from its own pre‐fire baseline as the response variable, with period (pre‐fire, smoke, fire, post‐fire) and burn severity as fixed effects and individual identity as a random intercept. Burn severity was calculated as the mean value within 100 m of each possum's refuge tree on the day of fire exposure, extracted from a Sentinel‐2‐derived dNBR severity raster (1 = unburnt to 4 = high severity). The unexposed individual (150.084) was assigned a severity value of 0, reflecting that it experienced no fire event at all; as it was never exposed, it contributed only to the pre fire and smoke‐period comparison. Models were fitted using the lme4 package (Bates et al. 2015). Effects are reported as estimates with 95% confidence intervals; effects on the log scale (VeDBA and step length) are back‐transformed to ratios (exp(*β*)), with confidence limits exponentiated from the log scale.

#### 2.6.2 Spatial

To assess post‐fire space use and habitat selection, we conducted two analyses using VHF tracking locations. First, we compared straight‐line distances moved between consecutive refuge locations before and after fire exposure for all exposed individuals, using only locations recorded 1 day apart so that distances were directly comparable without adjusting for elapsed time (53 one‐day steps, of which 48 had a severity value at the destination and were used in the model). We used a generalised linear mixed model (GLMM) with a Gamma distribution and log link, with period (pre‐ vs. post‐fire) and burn severity at the destination location (centred at Class 1, unburnt) as fixed effects, as the factors whose effects we tested, and individual possum as a random intercept because the possums are a sample from a wider population and each contributed repeated, non‐independent movements. The unexposed individual (150.084) was included as an additional reference point, with its movements pooled into the pre‐fire period as a measure of undisturbed baseline movement. Coefficients are on the log scale; exp(*β*) gives the multiplicative change in daily movement.

Second, we used a resource selection function (RSF) to test whether possums selected or avoided more severely burnt habitat post‐fire. We used a used–available design with availability defined separately for each location (Manly et al. 2002): for each post‐fire refuge location (used point), 10 random locations were generated within a 200 m radius to represent available but unchosen refuges. Burn severity (1 = unburnt to 4 = high severity) was extracted at each used and random location from a Sentinel‐2‐derived dNBR severity raster; points outside the mapped area were excluded (43 used and 401 available points were retained). We fitted a logistic generalised linear mixed model of used versus available with burn severity as a fixed effect and individual possum as a random intercept, so the coefficient describes relative selection (use relative to availability) at the population level, not preference or choice. Because this tests a single hypothesised covariate specified a priori, rather than selecting among many candidate covariates (Whittingham et al. 2006), support for selection was assessed by comparing this model with a null model (intercept and random intercept only) using Akaike's information criterion (AIC); the odds ratio per severity class is reported with its 95% confidence interval. The unexposed individual (150.084) was excluded from this analysis.

Model assumptions were assessed using residual diagnostics (residual‐vs.‐fitted plots, normal Q–Q plots, and tests of residual normality and autocorrelation; Figure S13). Strong autocorrelation between consecutive 1‐min observations in the GAM (lag‐1 *r* = 0.73) was accounted for with AR(1) errors, after which residual autocorrelation was negligible (lag‐1 *r* = −0.08), although the GAM residuals remained heavy‐tailed. Departures from normality in the step‐length and nocturnal burn‐severity models were mild.

## 3 Results

Possums used 34 refuges in total, averaging 4.4 dens per individual (±1.51 SD, range: 3–7). Before burn commencement on 4 May, possums were VHF‐tracked to their day refuges on three occasions, except for one individual (150.156), which was tracked once because it was collared later. This yielded 25 pre‐fire daytime refuge locations, 21 (hollows = 18, dreys = 3) of which were within the burn area. Six of these refuges were used by multiple adult possums; the joey associated with female 150.0362 was excluded from this count. Prior to the burn, refuges were most frequently located in sheoaks (*n* = 9), followed by jarrah (*n* = 5), marri (*n* = 4), grass trees (*n* = 2) and tuart (*n* = 1). Hollows were an average of 5 m (±1.92 SD, range: 2–12 m) above the ground, while dreys were an average of 2.9 m above the ground (±1.14 SD, range: 1.8–4.5 m). All possums remained within the burn cell during this time, except for possum 150.084 (F), which moved 110 m outside the burn cell on 1 May and remained there for the duration of the study. This left eight collared individuals, plus the uncollared joey, within the burn cell at the time of ignition.

Because the burn took 2 days to cover the entire cell, possum exposure to fire was staggered: four individuals were exposed on 4 May and five (including the joey) on 5 May (Table 1; Figure S12). On the day each possum was exposed to fire, eight of the nine possums (including the joey) were located in hollows within sheoaks, an average of 3.2 m above the ground (±0.81 SD, range: 2–4 m; Figure 2). The only possum not using a sheoak was located in a tuart, at least 6 m above the ground in a hollow. Evidence of the burn was visible within 1 m of all refuge trees except the tuart. Five of the eight refuge trees were directly impacted by fire, with scorch marks visible between 1 and 5 m above the ground (Figures S8–S11). The structural integrity of three of these trees was affected, with evidence of limb loss and trunk decay. None of the trees used by possums during the fire had been treated prior to the burn, that is, had leaf litter removed from around their bases. Individual possums experienced a mean burn severity of 1.93 (±0.21 SD, range: 1.43–2.10) at their fire‐day refuge, consistent with the low‐ to moderate‐severity classification of the burn overall.

**TABLE 1:** Summary of western ringtail possums (*Pseudocheirus occidentalis*) collared at Yalgorup National Park, May 2026. Refuge attributes refer to the daytime refuge location occupied at the time of fire exposure. Scorch height and closest burn evidence were recorded at the refuge tree following the prescribed burn.

Collar	Sex	Capture date	Days collared	Capture weight (g)	In burn cell	Refuge tree	Refuge type	Refuge height (m)	Closest burn evidence (m)	Scorch height (m)
150.022	M	29‐Apr	14	970	Yes	*A. fraseriana*	Hollow	2	0	3
150.036	F	30‐Apr	13	890	Yes	*A. fraseriana*	Hollow	4	0	3
150.0362	F	29‐Apr	14	960	Yes	*A. fraseriana*	Hollow	2.5	0	5
150.084	F	29‐Apr	14	1070	No	—	—	—	—	—
150.098	F	30‐Apr	20	890	Yes	*A. fraseriana*	Hollow	3	0	1
150.111	M	29‐Apr	14	750	Yes	*A. fraseriana*	Hollow	4	1	0
150.123	M	29‐Apr	14	1070	Yes	*E. gomphocephala*	Hollow	7.5	4	0
150.136	F	29‐Apr	14	1000	Yes	*A. fraseriana*	Hollow	4	0	3
150.156	M	2‐May	11	960	Yes	*A. fraseriana*	Hollow	3	1	0

**FIGURE 2:**
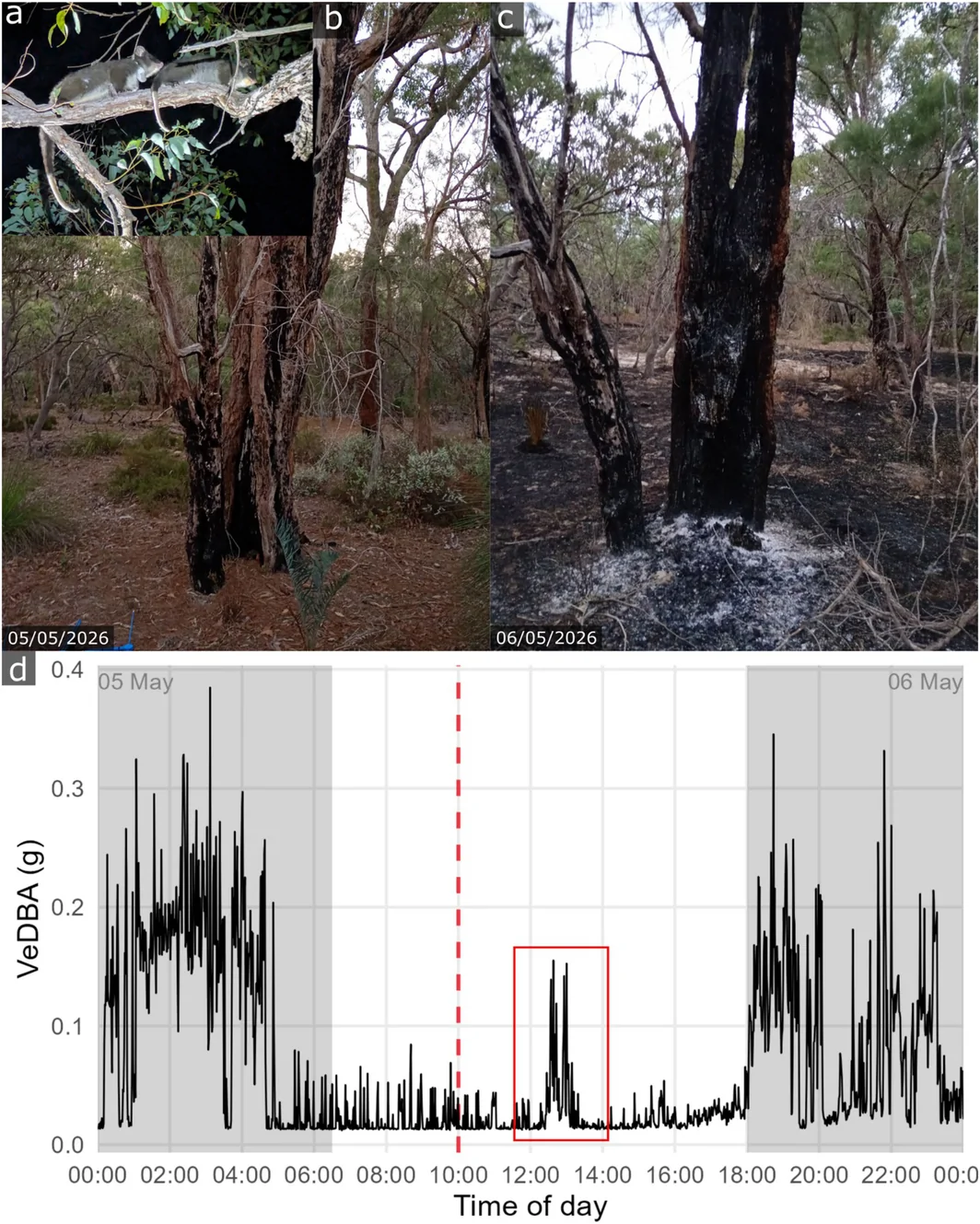
Refuge use and activity of a western ringtail possum (*Pseudocheirus occidentalis*) and its joey during a prescribed burn at Yalgorup National Park. (a) The focal female (150.0362) and her 650 g joey following the burn. (b) The refuge tree used on the morning of 5 May 2026, before fire reached the site. Both individuals were located in a hollow, approximately 2.5 m off the ground (c) The tree on 6 May 2026, showing scorch (0–5 m) and ground‐level charring following fire passage. (d) Fine‐scale activity (VeDBA, g) of the adult from 00:00 on 5 May to 00:00 on 6 May, with the red dashed line indicating the time of ignition at the refuge location and the red box highlighting a pronounced spike in activity during the fire period. Both the adult and joey were later visually confirmed to have survived the fire.

All nine possums (including the joey) within the burn cell were successfully VHF‐tracked on the morning immediately following fire exposure. Eight possums had moved to new locations, with movements averaging 181 m (±113.3 SD, range: 55–396 m). The only possum that did not move was the individual located in the tuart. All possums were confirmed to be alive on the 7th of May, when tracked during the night. Based on these observations, there was no evidence that any of the possums had been injured by the fire. Possums were tracked to daytime locations for a final time on the 11th of May. All possums were recaptured for collar removal between 13 and 20 May. Physical inspections revealed no evidence of injury, fire‐related or otherwise. Average change in body mass between initial capture and recapture was −0.1% (±5.83 SD, range: −6.8% to +9.5%). Of the 21 refuges used that were within the burn area prior to the burn, 10 (48%) were reused by possums in the days following fire passage. A further seven (33%) pre‐fire refuges remained structurally intact and potentially usable but were not selected again during the monitoring period. Four refuges (19%), including one grass tree and three sheoaks, were rendered unusable post‐fire, with fire damage compromising the structure of the refuge to the extent that it would no longer provide the same level of protection as before the burn.

Including period (period‐specific diel curves and period effects) improved the model (ΔAIC = 103 relative to a single shared diel curve). The smoke and fire periods were characterised by markedly reduced smooth amplitudes, indicating suppressed activity across much of the nocturnal period (Figure 3, Table 2). Suppression was most pronounced during the first part of the evening.

**FIGURE 3:**
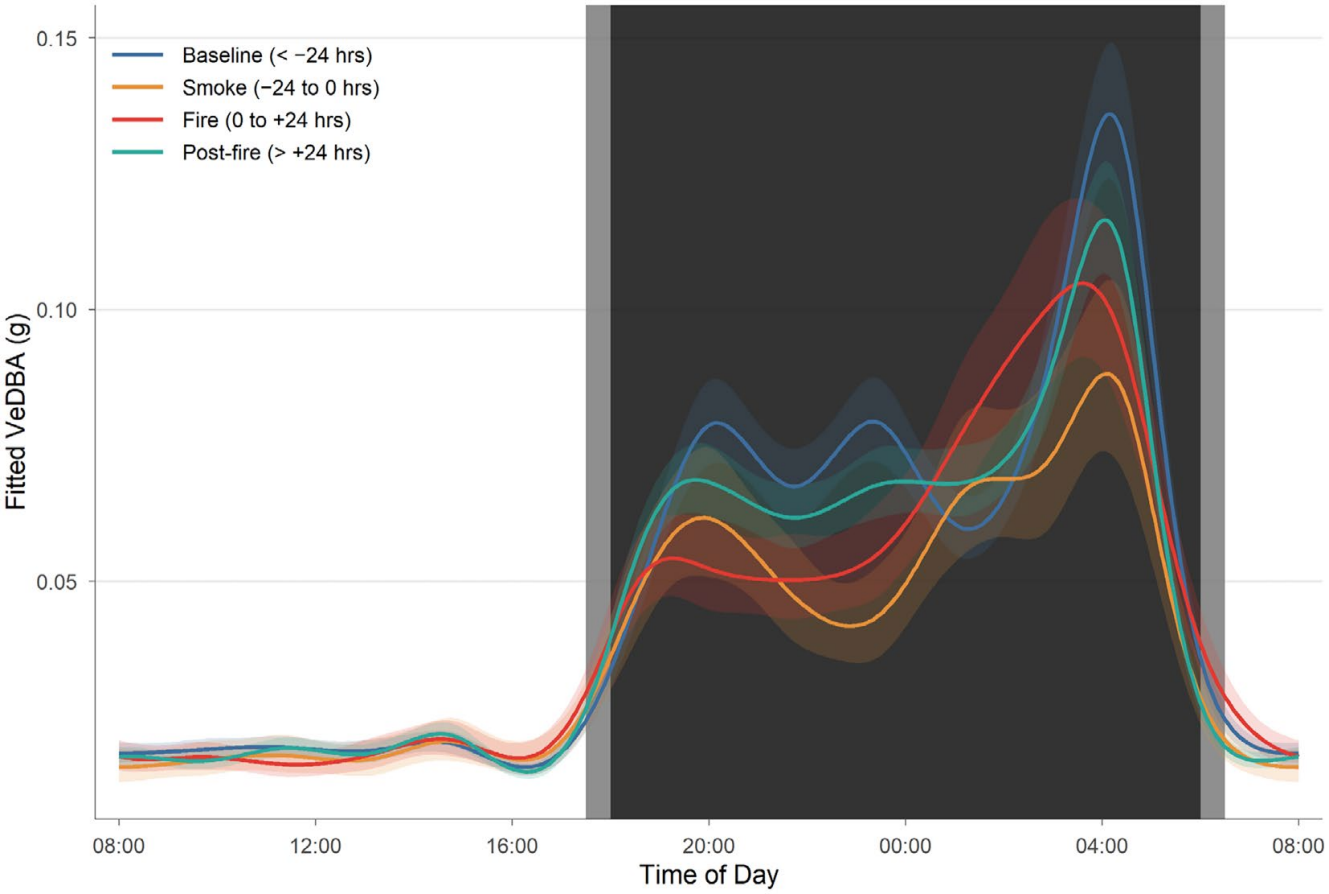
Diel activity patterns of western ringtail possums (*Pseudocheirus occidentalis*) across burn periods, estimated from vectorial dynamic body acceleration (VeDBA). Lines show GAM‐fitted VeDBA (g), back‐transformed from the log scale, and shaded bands show 95% confidence intervals (also back‐transformed). Grey shading indicates twilight and black shading indicates night.

**TABLE 2:** Generalised additive model (GAM) fitted to 1‐min mean VeDBA of western ringtail possums (*Pseudocheirus occidentalis*) across four fire‐related periods.

Term	Estimate	SE	95% CI
Intercept	−3.27	0.02	−3.31 to −3.24
Period (smoke)	−0.16	0.03	−0.21 to −0.10
Period (fire)	−0.07	0.02	−0.11 to −0.02
Period (post‐fire)	−0.05	0.01	−0.08 to −0.02
Sex (male)	0.03	0.03	−0.02 to 0.08

Term	edf	Ref.df	*F*
Time‐of‐day × Period (pre‐fire)	12.61	13	330.74
Time‐of‐day × Period (smoke)	11.06	13	43.83
Time‐of‐day × Period (fire)	11.05	13	80.65
Time‐of‐day × Period (post‐fire)	12.70	13	354.30
Individual (random smooth)	4.98	7	2.63

*Note:* Parametric terms are on the log scale, with the baseline (pre‐fire, female) as the reference level; standard errors and 95% confidence intervals are shown.

Abbreviation: edf, effective degrees of freedom.

Similarly, the LMM predictions showed clear reductions in overall activity during smoke exposure and fire when compared to the pre‐fire baseline (Figure 4, Table 3). Nocturnal activity was approximately 19% lower during smoke (0.093 vs. 0.115) and 14% lower during the fire period (0.099 vs. 0.115), with a partial recovery post‐fire (0.107; around 7% below baseline). Diurnal changes were small and uncertain (Table 3). Burn severity did not clearly modify the magnitude of these responses (95% CIs include 0; Table 4).

**FIGURE 4:**
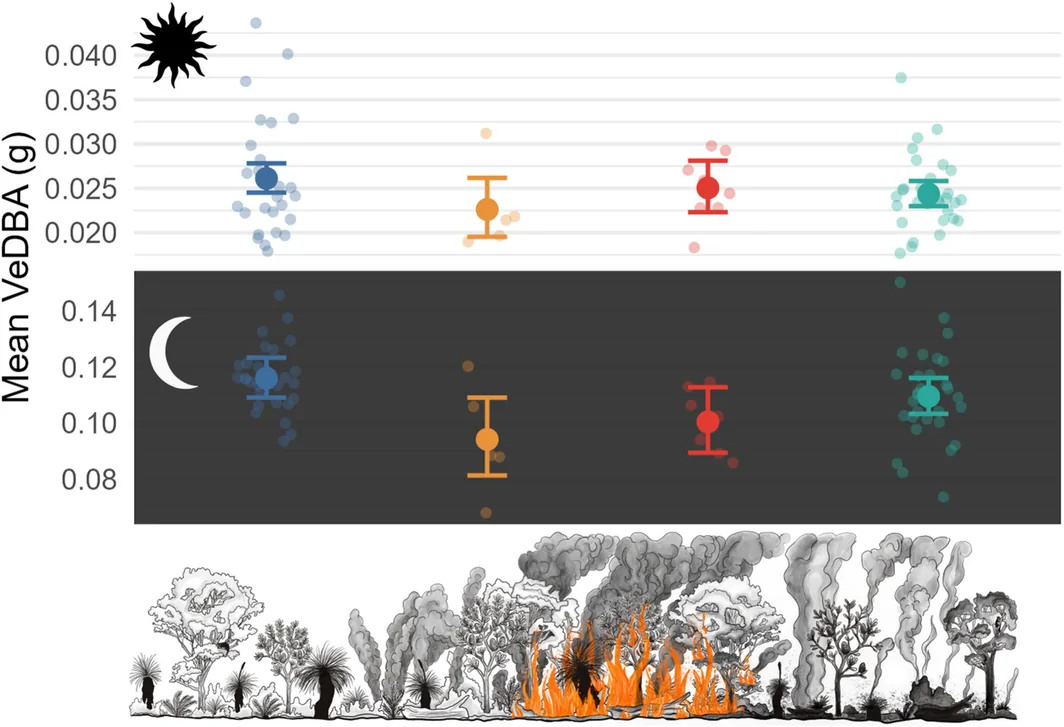
VeDBA (g) of western ringtail possums (*Pseudocheirus occidentalis*) during diurnal (top) and nocturnal (bottom) periods across four fire‐related periods. Points show per‐possum per‐night means; larger points and error bars show predicted values with 95% confidence intervals from linear mixed models (LMMs) fitted separately for diurnal and nocturnal activity to log‐transformed VeDBA and back‐transformed for plotting. Periods are baseline (blue), smoke exposure (orange), fire (red) and post‐fire (teal). Artwork by Shelby Middleton.

**TABLE 3:** Fixed effects from linear mixed models (LMMs) fitted separately to diurnal and nocturnal mean VeDBA of western ringtail possums (*Pseudocheirus occidentalis*) across four fire‐related periods. Models used a Gaussian distribution and individual possum as a random intercept.

Term	Diurnal			Nocturnal		
	Estimate	SE	95% CI	Estimate	SE	95% CI
Intercept	−3.67	0.04	−3.76 to −3.58	−2.16	0.03	−2.23 to −2.10
Period (smoke)	−0.14	0.09	−0.32 to 0.04	−0.21	0.06	−0.33 to −0.10
Period (fire)	−0.02	0.07	−0.17 to 0.13	−0.15	0.05	−0.25 to −0.06
Period (post‐fire)	−0.05	0.05	−0.14 to 0.05	−0.07	0.03	−0.13 to −0.01

*Note:* Estimates are on the log scale, with standard errors and 95% confidence intervals.

**TABLE 4:** Fixed effects from linear mixed models testing whether burn severity influenced the size of the activity response to fire, fitted separately for diurnal and nocturnal deviation in log(VeDBA) from each possum's own pre‐fire baseline.

Term	Diurnal			Nocturnal		
	Estimate	SE	95% CI	Estimate	SE	95% CI
Intercept (pre‐fire)	0.03	0.11	−0.20 to 0.26	−0.05	0.05	−0.16 to 0.06
Period (smoke)	−0.14	0.09	−0.32 to 0.04	−0.19	0.06	−0.31 to −0.08
Period (fire)	0.00	0.07	−0.14 to 0.15	−0.16	0.05	−0.25 to −0.06
Period (post‐fire)	−0.03	0.05	−0.12 to 0.07	−0.08	0.03	−0.14 to −0.02
Burn severity	−0.01	0.06	−0.14 to 0.11	0.03	0.03	−0.03 to 0.09

*Note:* Models included period and burn severity as fixed effects, with individual possum as a random intercept. Estimates are on the log scale, with standard errors and 95% confidence intervals.

Step length did not differ between pre‐ and post‐fire periods and was not clearly related to burn severity (Figure 5a, Table 5). Used and available locations had similar burn severity, and the model with burn severity did not improve on the null model (Figure 5b, Table 6).

**FIGURE 5:**
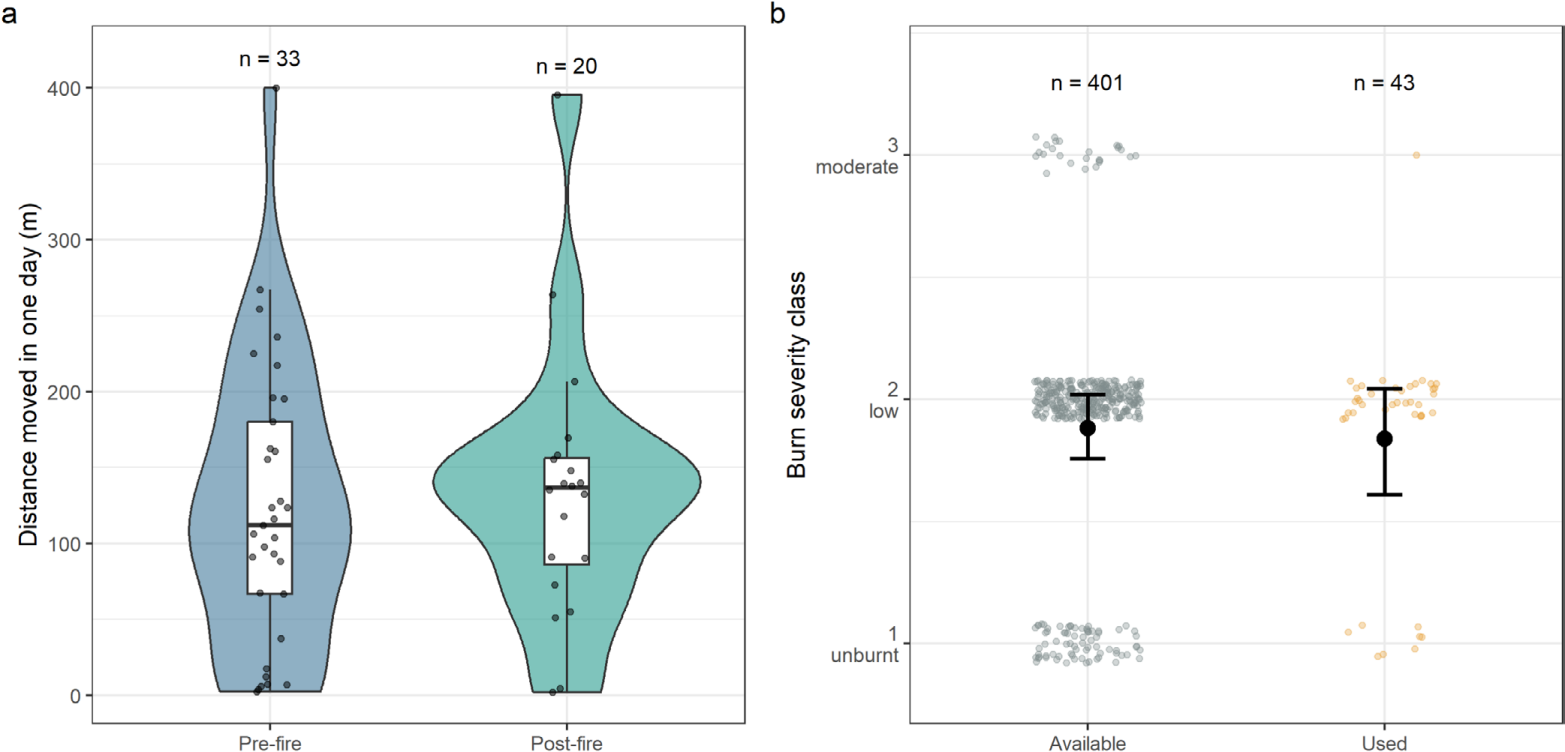
Space use of western ringtail possums (*Pseudocheirus occidentalis*) before and after a prescribed burn at Yalgorup National Park, May 2026. (a) Distance moved between fixes 1 day apart before and after fire exposure; points are individual steps. (b) Burn severity class at used refuge locations and at available locations (random points within 200 m of each used location); points are individual locations (jittered) and black symbols show the mean with a 95% bootstrap confidence interval (resampling individuals).

**TABLE 5:** Gamma GLMM (log link) of distance moved between fixes 1 day apart, comparing pre‐ and post‐fire periods, with burn severity at the destination fix (centred at Class 1, unburnt) as an additional fixed effect and a random intercept for individual.

Term	Estimate	SE	95% CI
Intercept (pre‐fire, unburnt)	5.08	0.37	4.36 to 5.81
Post‐fire	0.04	0.25	−0.45 to 0.54
Burn severity (destination, per class)	−0.31	0.34	−0.98 to 0.36

*Note:* Estimates are on the log scale, with standard errors and 95% confidence intervals.

**TABLE 6:** Candidate logistic GLMMs of used versus available refuge locations, each with an individual random intercept, compared using Akaike's information criterion (AIC), ΔAIC (difference from the lowest AIC) and Akaike weights.

Resource selection (logistic GLMM)	Parameters	AIC	ΔAIC	Akaike weight
Null (intercept only)	2	286.47	0.00	0.70
Burn severity	3	288.13	1.66	0.30

*Note:* The null model is intercept only; parameters are the number of fixed effects plus the individual variance.

## 4 Discussion

Climate change is intensifying wildfire risk globally (Jones et al. 2022), and prescribed burning remains one of the most frequently used tools for managing that risk (Gordon et al. 2025). However, the direct influence of prescribed fire on wildlife remains poorly understood (Jolly et al. 2022). Here, we show that a Critically Endangered possum, widely regarded as highly vulnerable to fire (Doherty 2026), can survive low‐ to moderate‐severity prescribed fire and its immediate aftermath. We also found the majority (81%) of refuges used prior to the burn were still available to be used after the burn. Our findings do not imply that ngwayir can survive all fire events, nor that prescribed fire has no longer‐term effects on individuals or populations, but that the acute impacts of low‐ to moderate‐severity prescribed fire may be lower than previously assumed. Our work adds to growing evidence that animals can exhibit surprisingly high survival through fire events, particularly where fire severity is low to moderate and sufficient refuges are available (Jolly et al. 2022).

We found that all eight collared individuals exposed to fire, plus the uncollared joey, survived direct exposure, despite several refuges being consumed by fire. This contradiction is best explained by considering the evolutionary and behavioural mechanisms that shape how animals perceive and respond to fire (Pausas and Parr 2018; Jones et al. 2023). Because fire represents an immediate and potentially lethal threat, many animal species in fire‐prone ecosystems are able to detect smoke, fire sounds and other fire‐related cues, and to respond with behaviours that reduce exposure and increase the likelihood of survival (Nimmo et al. 2021). The survival of all nine exposed possums suggests that ngwayir could possess such cue‐based avoidance capacities, and that these behaviours were effective during a low severity prescribed burn. Our findings add to a growing body of evidence that animals have complex behavioural responses to fire that foster survival. For example, sleepy lizards (*Tiliqua rugosa*; Jolly et al. 2025), Mediterranean skinks (*Psammodromus algirus*; Álvarez‐Ruiz et al. 2023) and reed frogs ( *Hyperolius nitidulus* ) respond to fire cues (smoke and fire sounds) by attempting to flee (Grafe et al. 2002), and Gould's long‐eared bats ( *Nyctophilus gouldi* ) show immediate physiological arousal when exposed to smoke (Doty et al. 2018).

While there is very little known about how animals behaviourally respond to fire cues in the wild (Michel et al. 2023), our accelerometry data provides some unique insights. First, we found that possum activity tended to decrease after ignition, when smoke cues would have been signalling fire's presence but the fire was distant enough to not represent a substantial threat. This reduction in activity could be indicative of heightened vigilance or preparatory behavioural adjustment (Nowack et al. 2018; Nimmo et al. 2021). However, the day following the fire, all individuals had moved from their pre‐fire refuges, on average 181 m away, presumably to avoid the fire as it moved closer, and eventually consumed or scorched several of the refuge trees. We cannot determine how they escaped the oncoming fire, but ngwayir, being arboreal, are very capable climbers, able to rapidly traverse canopy vegetation (Baker and Gynther 2023). This form of escape would provide the benefit of avoiding some of the direct impacts of fire, which in low intensity burns are often concentrated at the ground level.

The capacity to detect and respond to fire cues does not guarantee survival, particularly when fires are intense, severe and fast moving. For example, in northern Australia, frill‐neck lizards ( *Chlamydosaurus kingii* ) climb higher into trees as fire approaches, a behaviour that improves survival during low‐severity fires (Griffiths and Christian 2006; Nimmo et al. 2021). Under more intense fires, however, this response can place individuals in the flame zone, causing mortality (Griffiths and Christian 2006). The same could be true for ngwayir behaviours that improve survival during low‐to‐moderate severity fire but could be overwhelmed under more severe, fast‐spreading fires with higher flame heights.

In our case study, several features of the prescribed burn and fire conditions likely played a crucial role in enabling possums to avoid injury or death. The fire progressed slowly (18–30 m/h) under mild temperatures, light winds and relatively high humidity, and ignition crews avoided lighting highly combustible vegetation such as grass trees that would increase fire intensity. Additional precautions, such as removing leaf litter from the bases of hollow‐bearing trees, further reduced the likelihood of hollows igniting. These factors created a context in which possums had the time to relocate safely once they detected fire. The vegetation within the study area is largely in good condition, providing a well‐connected canopy that probably facilitated efficient arboreal movement. Should fire occur in areas with less well‐connected canopies, possums could be forced to descend to the ground to relocate between trees or refuges, increasing their exposure to flames and radiant heat and thereby reducing their likelihood of surviving a burn.

The fire regimes experienced by ngwayir in recent millennia were shaped for much of that time by Noongar peoples' cultural burning practices. Historical accounts, explorer diaries and contemporary Noongar knowledge indicate that burning was often localised, seasonal and governed by rights and responsibilities to Country, with fire used to maintain open grassy areas, renew plant foods, attract game and create fine‐grained vegetation mosaics (Lullfitz et al. 2017). In coastal and near‐coastal areas with regular Aboriginal occupation, including parts of the Swan Coastal Plain between Busselton and the Swan River, Hassell and Dodson (2003) inferred short fire intervals from early historical observations of Aboriginal burning and historical and archaeological synthesis. Contemporary work with Noongar Elders further emphasises that Noongar fire knowledge is highly place‐specific, with appropriate burning depending on vegetation, fuel, weather, cultural values and Noongar governance (Rodrigues et al. 2022). Further engagement with Noongar people is required to better understand the fire regimes experienced by ngwayir prior to European colonisation, and the ecological context within which the species persisted over recent millennia.

Despite occurring in structurally similar habitat < 2 km apart, our results differ substantially from those reported by Zylstra (2023). There are multiple potential explanations for this. One is that fire intensity or severity was higher in the burn studied by Zylstra (2023) than in the current study, reducing the opportunity for possums to escape. However, a direct comparison is complicated because it is unclear which fire Zylstra (2023) actually studied. Zylstra (2023) states that the fire he studied was a prescribed burn at Warrungup Spring Reserve on May 16 in autumn of 2018, but there is no record of a prescribed burn being undertaken in that reserve on that date. The City of Mandurah did undertake a prescribed burn in Warrungup Spring Reserve, but in the spring of 2016. Despite this uncertainty, assuming that the prescribed fire that Zylstra (2023) described was the 2016 burn in Warrungup Spring Reserve, the reported fire behaviour of that fire appears broadly consistent with that observed in the current study. Both Zylstra (2023) and the City of Mandurah describe the Warrungup Spring burn as a slow‐moving, low‐intensity fire with generally low flame heights and occasional torching of grass tree skirts. This is similar to the fire behaviour recorded in the current study, even though the Warrungup Spring burn occurred under substantially lower fuel loads, approximately 6 t/ha (Zylstra 2023), compared with 10–23 t/ha in the current study. This suggests that fire behaviour alone is unlikely to explain the markedly different mortality outcomes between the two studies.

It is also possible that differences in individual history contributed to the contrasting outcomes. Five possums in Zylstra's study had been rehabilitated and released, and relocation or prior trauma could plausibly affect their ability to detect and respond to early fire cues. This explanation remains speculative, however, because Zylstra (2023) does not report which individuals were rehabilitated or whether their fates differed from those of wild‐born animals. A further possibility is that the difference reflects how fates were assigned. In our study, individual fates were confirmed using VHF collars, reducing uncertainty around survival outcomes. By contrast, the reported 77% mortality in Zylstra (2023) was inferred from an unspecified number of burnt remains, flies near inaccessible hollows and the inability to relocate individuals after the fire. This creates greater uncertainty around individual fates, which is why a recent global synthesis of animal survival during fire included only animals whose fates were confirmed by tracking (Jolly et al. 2022).

Our findings come with some important caveats. First, our study involved only nine possums in a single prescribed burn. Although our sampling effort is comparable to other studies of animal survival during fire (Jolly et al. 2022), greater replication would strengthen inference, given that prescribed burns vary widely in intensity, severity and coverage due to differences in vegetation type, structure, condition and age, as well as burn location, season and lighting pattern. While this burn achieved close to the 70% coverage target set by DBCA, it was accompanied by several mitigation measures—including removing leaf litter from the bases of hollow‐bearing trees and avoiding the direct ignition of grass trees—which may have reduced the likelihood of possum injury compared with burns where such measures are not applied. In addition, our results reflect only immediate survival during a low‐severity fire; the study was not designed to assess delayed mortality, long‐term sublethal effects or population‐level responses. Finally, individuals sometimes shared refuges, so social interactions and any behavioural effects of wearing a collar may have contributed variation that we could not quantify.

Our study provides rare individual‐level evidence of how a critically endangered arboreal mammal responds to prescribed fire. First, all nine fire‐exposed ngwayir (eight collared, one uncollared joey) survived direct exposure to a low‐severity burn, even where refuge trees were scorched or structurally compromised. Second, post‐fire movements and refuge selection showed little deviation from pre‐fire patterns, indicating limited disruption to space use at the home‐range scale. Third, accelerometry revealed clear, short‐term shifts in diel activity during smoke and fire exposure, including suppressed nocturnal activity and elevated diurnal movement around the time of fire passage. Taken together, these behavioural responses are consistent with traits shaped by long‐term exposure to frequent, low‐severity fires, potentially contributed to in part by Noongar cultural burning. As climate change increases the risk that wildlife will be exposed to more severe and unpredictable fire events, identifying the thresholds beyond which these behavioural strategies cease to provide protection will be essential for designing prescribed burning programs that mitigate hazard while maintaining the persistence of threatened species.

## Author Contributions

**Harry Moore:** investigation (lead), project administration (lead), writing – original draft (lead), writing – review and editing (lead). **Shelby Middleton:** conceptualization (equal), investigation (equal), methodology (equal), project administration (equal), writing – original draft (equal), writing – review and editing (equal). **Dale Nimmo:** writing – original draft (equal), writing – review and editing (equal). **Ciara McIlduff:** conceptualization (equal), investigation (equal), project administration (equal), writing – original draft (equal), writing – review and editing (equal). **Nicole Willers:** conceptualization (equal), investigation (equal), project administration (equal), writing – original draft (equal), writing – review and editing (equal).

## Funding

Funding for this study was provided by the Department of Biodiversity, Conservation and Attractions.

## Conflicts of Interest

The authors declare no conflicts of interest.

## Supporting information


**Figure S1:** Pre‐fire vegetation within the SWC_118 prescribed burn cell at Yalgorup National Park, Western Australia.
**Figure S2:** Pre‐fire vegetation within the SWC_118 prescribed burn cell at Yalgorup National Park, Western Australia.
**Figure S3:** Pre‐fire vegetation within the SWC_118 prescribed burn cell at Yalgorup National Park, Western Australia.
**Figure S4:** Fire moving through the SWC_118 prescribed burn cell at Yalgorup National Park, Western Australia.
**Figure S5:** Fire moving through the SWC_118 prescribed burn cell at Yalgorup National Park, Western Australia.
**Figure S6:** Post‐fire habitat conditions within the SWC_118 prescribed burn cell at Yalgorup National Park, Western Australia.
**Figure S7:** Area most impacted by the SWC_118 prescribed burn, due to high density of grass trees, at Yalgorup National Park, Western Australia.
**Figure S8:** Refuge tree used by possum 150.022 during the passage of fire within the SWC_118 prescribed burn cell at Yalgorup National Park, Western Australia.
**Figure S9:** Refuge tree used by possum 150.0362 during the passage of fire within the SWC_118 prescribed burn cell at Yalgorup National Park, Western Australia.
**Figure S10:** Refuge tree used by possum 150.136 during the passage of fire within the SWC_118 prescribed burn cell at Yalgorup National Park, Western Australia.
**Figure S11:** Refuge tree used by possum 150.036 during the passage of fire within the SWC_118 prescribed burn cell at Yalgorup National Park, Western Australia.
**Figure S12:** Fine‐scale activity (VeDBA, g) of all nine western ringtail possums (*Pseudocheirus occidentalis*) on their day of fire exposure, including the unexposed control (150.084). Red dashed lines indicate the time of ignition (10:00) at each possum's refuge location on its own fire day; grey shading indicates night.
**Figure S13:** Residual diagnostics for the linear mixed models and the step‐length model. Left: standardised residuals versus fitted values, with the red dashed line at zero. Right: normal Q–Q plots, with the red line showing the expected normal quantiles. Rows show the Table 3 diurnal and nocturnal models, the Table 4 diurnal and nocturnal models, and the Table 5 step‐length model; the activity models were fitted to log‐transformed VeDBA.

## Data Availability

The data and R code used in this study are available at: https://doi.org/10.5061/dryad.qz612jmxt.

## References

[ece374637-bib-0001] Álvarez‐Ruiz, L. , J. G. Pausas , D. T. Blumstein , and B. J. Putman . 2023. “Lizards' Response to the Sound of Fire Is Modified by Fire History.” Animal Behaviour 196: 91–102. 10.1016/j.anbehav.2022.12.002.

[ece374637-bib-0002] Archibald, R. , B. Bowen , G. S. J. Hardy , J. Fox , and D. Ward . 2005. “Changes to Tuart Woodland in Yalgorup National Park Over Four Decades.” In A Forest Conscienceness: Proceedings 6th National Conference of the Australian Forest History Society Inc, 363–372. Millpress Science Publishers.

[ece374637-bib-0003] Baker, A. , and I. Gynther . 2023. Strahan's Mammals of Australia. 4th ed. Bloomsbury Publishing.

[ece374637-bib-0049] Bates, D. , M. Mächler , B. Bolker , and S. Walker . 2015. “Fitting Linear Mixed‐Effects Models Using lme4.” Journal of Statistical Software 67, no. 1. 10.18637/jss.v067.i01.

[ece374637-bib-0004] Burbidge, A. A. , and J. Zichy‐Woinarski . 2017. “*Pseudocheirus occidentalis* .” The IUCN Red List of Threatened Species 2017. 10.2305/IUCN.UK.2017-3.RLTS.T18492A21963100.en.

[ece374637-bib-0005] Bureau of Meteorology . 2026. “Mandurah, Western Australia: July 2026 Daily Weather Observations (Product IDCJDW6077).” https://www.bom.gov.au/climate/dwo/IDCJDW6077.latest.shtml.

[ece374637-bib-0006] Cunningham, C. X. , G. J. Williamson , and D. M. Bowman . 2024. “Increasing Frequency and Intensity of the Most Extreme Wildfires on Earth.” Nature Ecology & Evolution 8: 1420–1425. 10.1038/s41559-024-02452-2. 38914710

[ece374637-bib-0007] DBCA . 2026. “DBCA Fire History (DBCA‐060).” https://catalogue.data.wa.gov.au/dataset/dbca‐fire‐history.

[ece374637-bib-0008] DBCA , and DFES . 2020. Prescribed Burning Implementation Guide. Operational Guide Version 3.0. Government of Western Australia.

[ece374637-bib-0009] DCCEEW . 2026. “Threatened Species Under the EPBC Act.” https://www.dcceew.gov.au/environment/biodiversity/threatened/species.

[ece374637-bib-0010] Department of Parks and Wildlife . 2017. “Western Ringtail Possum (*Pseudocheirus occidentalis*) Recovery Plan.” Wildlife Management Program No. 54. Department of Parks and Wildlife Perth, WA, Perth, Western Australia. https://www.dcceew.gov.au/sites/default/files/documents/recovery‐plan‐western‐ringtail‐possum.pdf.

[ece374637-bib-0011] Doherty, T. S. 2026. “Fire Responses of Fauna in South‐Western Australia: Clarifying Some Recent Assertions.” Australian Journal of Zoology 74: ZO25049. 10.1071/ZO25049.

[ece374637-bib-0012] Doherty, T. S. , M. G. Allen , K. Bain , et al. 2026. “Animal Taxa Threatened by Adverse Fire Regimes in Western Australia: A Synthesis and Outlook.” Pacific Conservation Biology 32: PC25051. 10.1071/pc25051.

[ece374637-bib-0013] Doty, A. C. , S. E. Currie , C. Stawski , and F. Geiser . 2018. “Can Bats Sense Smoke During Deep Torpor?” Physiology & Behavior 185: 31–38. 10.1016/j.physbeh.2017.12.019. 29253491

[ece374637-bib-0014] Driscoll, D. A. , K. J. Macdonald , R. K. Gibson , et al. 2024. “Biodiversity Impacts of the 2019–2020 Australian Megafires.” Nature 635: 898–905. 10.1038/s41586-024-08174-6 PMC11602714 39537920

[ece374637-bib-0015] Fenner, A. , and C. M. Bull . 2007. “Short‐Term Impact of Grassland Fire on the Endangered Pygmy Bluetongue Lizard.” Journal of Zoology 272: 444–450.

[ece374637-bib-0016] Gordon, L. , M. J. Evans , P. Zylstra , and D. B. Lindenmayer . 2025. “Trends and Gaps in Prescribed Burning Research.” Environmental Management 75: 746–760. 10.1007/s00267-025-02119-z. PMC11965264 39890629

[ece374637-bib-0017] Grafe, T. U. , S. Doebler , and K. E. Linsenmair . 2002. “Frogs Flee From the Sound of Fire.” Proceedings of the Royal Society of London. Series B: Biological Sciences 269: 999–1003. 10.1098/rspb.2002.1974 PMC1690992 12028755

[ece374637-bib-0018] Griffiths, A. , and K. Christian . 2006. “The Effects of Fire on the Frillneck Lizard ( *Chlamydosaurus kingii* ) in Northern Australia.” Australian Journal of Ecology 21: 386–398. 10.1111/j.1442-9993.1996.tb00625.x.

[ece374637-bib-0019] Hassell, C. W. , and J. R. Dodson . 2003. “Fire History of South‐West Western Australia Prior to European Settlement in 1826–1829.” In Fire in Ecosystems of South‐West Western Australia: Impacts and Management, edited by I. Abbott and N. Burrows , 71–85. Backhuys Publishers.

[ece374637-bib-0020] Inions, G. , M. Tanton , and S. Davey . 1989. “Effect of Fire on the Availability of Hollows in Trees Used by the Common Brushtail Possum, *Trichosurus vulpecula* Kerr, 1792, and the Ringtail Possum, *Pseudocheirus peregrinus* Boddaerts, 1785.” Wildlife Research 16: 449–458. 10.1071/WR9890449.

[ece374637-bib-0021] IPCC . 2022. Climate Change 2022: Impacts, Adaptation and Vulnerability. Contribution of Working Group II to the Sixth Assessment Report of the Intergovernmental Panel on Climate Change, edited by H.‐O. Pörtner , D. C. Roberts , M. Tignor , et al. Cambridge University Press. 10.1017/9781009325844.

[ece374637-bib-0022] Jolly, C. J. , C. R. Dickman , T. S. Doherty , et al. 2022. “Animal Mortality During Fire.” Global Change Biology 28: 2053–2065. 10.1111/gcb.16044 34989061

[ece374637-bib-0023] Jolly, C. J. , D. G. Nimmo , A. J. Carthey , E. H. Van De Pas , and M. J. Whiting . 2025. “From Anecdote to Evidence: Experimental Validation of Fire‐Cue Recognition in Australian Sleepy Lizards.” Biology Letters 21: 20250364. 10.1098/rsbl.2025.0364 PMC12440628 40957554

[ece374637-bib-0024] Jones, B. , R. How , and D. Kitchener . 1994. “A Field Study of Pseudocheirus Occidentalis (Marsupialia: Petauridae) II. Population Studies.” Wildlife Research 21: 189–201. 10.1071/wr9940189.

[ece374637-bib-0025] Jones, G. M. , J. F. Goldberg , T. M. Wilcox , et al. 2023. “Fire‐Driven Animal Evolution in the Pyrocene.” Trends in Ecology & Evolution 38: 1072–1084. 10.1016/j.tree.2023.06.003. 37479555

[ece374637-bib-0026] Jones, M. W. , J. T. Abatzoglou , S. Veraverbeke , et al. 2022. “Global and Regional Trends and Drivers of Fire Under Climate Change.” Reviews of Geophysics 60: e2020RG000726. 10.1029/2020RG000726.

[ece374637-bib-0027] Lullfitz, A. , J. Dortch , S. D. Hopper , C. Pettersen , and D. Guilfoyle . 2017. “Human Niche Construction: Noongar Evidence in Pre‐Colonial Southwestern Australia.” Conservation and Society 15: 201–216. 10.4103/cs.cs_16_75.

[ece374637-bib-0028] Manly, B. F. , L. L. McDonald , D. L. Thomas , T. L. McDonald , and W. P. Erickson . 2002. Resource Selection by Animals: Statistical Design and Analysis for Field Studies. Springer.

[ece374637-bib-0029] Michel, A. , J. R. Johnson , R. Szeligowski , E. G. Ritchie , and A. Sih . 2023. “Integrating Sensory Ecology and Predator–Prey Theory to Understand Animal Responses to Fire.” Ecology Letters 26: 1050–1070. 10.1111/ele.14231. 37349260

[ece374637-bib-0030] Moore, H. A. , N. Beerkens , R. J. Teale , R. Lymbery , and H. Kilian . 2026. “Sublethal Effects of Extreme Heat on a Critically Endangered Marsupial.” Pacific Conservation Biology 32: PC25052. 10.1071/PC25052.

[ece374637-bib-0031] Nimmo, D. G. , A. J. Carthey , C. J. Jolly , and D. T. Blumstein . 2021. “Welcome to the Pyrocene: Animal Survival in the Age of Megafire.” Global Change Biology 27: 5684–5693. 10.1111/gcb.15834. 34404117

[ece374637-bib-0032] Nowack, J. , C. Stawski , G. Körtner , and F. Geiser . 2018. “Physiological and Behavioral Responses of an Arboreal Mammal to Smoke and Charcoal–Ash Substrate.” Physiology & Behavior 184: 116–121. 10.1016/j.physbeh.2017.11.020. 29158126

[ece374637-bib-0033] Pausas, J. G. , and C. L. Parr . 2018. “Towards an Understanding of the Evolutionary Role of Fire in Animals.” Evolutionary Ecology 32: 113–125. 10.1007/s10682-018-9927-6.

[ece374637-bib-0034] Povh, L. F. , N. Willers , J. M. Shephard , and P. A. Fleming . 2023. “A Conservation‐Significant Threatened Mammal Uses Fire Exclusions and Shifts Ranges in the Presence of Prescribed Burning.” International Journal of Wildland Fire 32: 1291–1303. 10.1071/WF22196.

[ece374637-bib-0035] Qasem, L. , A. Cardew , A. Wilson , et al. 2012. “Tri‐Axial Dynamic Acceleration as a Proxy for Animal Energy Expenditure; Should We Be Summing Values or Calculating the Vector?” PLoS One 7: e31187. 10.1371/journal.pone.0031187. PMC3281952 22363576

[ece374637-bib-0051] R Core Team . 2024. “R: A Language and Environment for Statistical Computing.” R Foundation for Statistical Computing. https://www.R‐project.org/.

[ece374637-bib-0036] Rodrigues, U. , A. Lullfitz , L. Coyne , et al. 2022. “Indigenous Knowledge, Aspiration, and Potential Application in Contemporary Fire Mitigation in Southwest Australia.” Human Ecology 50: 963–980. 10.1007/s10745-022-00359-9.

[ece374637-bib-0037] Sneeuwjagt, R. , and G. Peet . 1998. Forest Fire Behaviour Tables. Department of Conservation and Land Management.

[ece374637-bib-0038] Teale, R. J. , J. M. Potts , P. Kendrick , et al. 2025. “After Expert Elicitation: The Importance of Evidence‐Based Conservation Assessments to Close Knowledge Gaps for Threatened Species Conservation.” Biological Conservation 308: 111237. 10.1016/j.biocon.2025.111237.

[ece374637-bib-0039] TechnoSmart . 2026. “Mammal Trek Remote: Axy5 Datalogger.” https://www.technosmart.eu/mammal‐trek‐remote/.

[ece374637-bib-0040] Turner, J. M. 2022. “Capturing Arboreal Mammals by Hook and Net.” In Wildlife Research in Australia: Practical and Applied Methods, edited by B. P. Smith , H. P. Waddley , C. C. Alberthsen , and J. O. Hampton , 130–133. CSIRO Publishing. https://www.publish.csiro.au/book/8134/.

[ece374637-bib-0041] van Dongen, R. , A. Deshayes , V. Densmore , B. Miller , and K. Ruthrof . 2023. “Fire Severity Mapping and Dense Forest Stands.” Department of Biodiversity, Conservation and Attractions, Perth, Western Australia.

[ece374637-bib-0042] van Eeden, L. M. , and C. R. Dickman . 2023. “Estimating the Number of Wild Animals Affected by Australia's 2019–20 Wildfires.”

[ece374637-bib-0043] Wayne, A. 2006. “Fire Management Guideline: Ngwayir (Western Ringtail Possum).” Science Division, Department of Conservation and Land Management, Manjimup, Western Australia.

[ece374637-bib-0044] Wayne, A. F. , A. Cowling , D. B. Lindenmayer , et al. 2006. “The Abundance of a Threatened Arboreal Marsupial in Relation to Anthropogenic Disturbances at Local and Landscape Scales in Mediterranean‐Type Forests in South‐Western Australia.” Biological Conservation 127: 463–476. 10.1016/j.biocon.2005.09.007.

[ece374637-bib-0045] Wayne, A. F. , M. Maxwell , J. Potts , A. Barrett , and B. Geary . 2022. “Ngwayir Associations With Management and Environmental Factors in the Upper Warren.” Department Biodiversity, Conservation and Attractions.

[ece374637-bib-0046] Wayne, A. F. , M. A. Maxwell , and N. D. Harrison . 2026. “Down but Not Out: Long‐Term Monitoring Reveals Possum Population Changes and Associations in the Southern Jarrah Forest.” Wildlife Research 53: WR26027. 10.1071/WR26027.

[ece374637-bib-0047] Whittingham, M. J. , P. A. Stephens , R. B. Bradbury , and R. P. Freckleton . 2006. “Why Do We Still Use Stepwise Modelling in Ecology and Behaviour?” Journal of Animal Ecology 75: 1182–1189. 10.1111/j.1365-2656.2006.01141.x. 16922854

[ece374637-bib-0050] Wood, S. N. 2000. “Modelling and Smoothing Parameter Estimation With Multiple Quadratic Penalties.” Journal of the Royal Statistical Society, Series B: Statistical Methodology 62, no. 2: 413–428. 10.1111/1467-9868.00240.

[ece374637-bib-0048] Zylstra, P. 2023. “Quantifying the Direct Fire Threat to a Critically Endangered Arboreal Marsupial Using Biophysical, Mechanistic Modelling.” Austral Ecology 48: 266–288. 10.1111/aec.13264.

