# Supplementary material for: Under Fire: High Survival Rates of a Critically Endangered Marsupial During a Low‐Severity Prescribed Burn
Source: Ecol Evol. 2026 Nov 5;16(11):e74637. doi: 10.1002/ece3.74637 (PMC13739421; doi:10.1002/ece3.74637)
Supplement: Supplementary file 1 [file ECE3-16-e74637-s001.docx]

Figure S1 - Pre‑fire vegetation within the SWC_118 prescribed burn cell at Yalgorup National Park, Western Australia.

Figure S2 - Pre‑fire vegetation within the SWC_118 prescribed burn cell at Yalgorup National Park, Western Australia.

Figure S3 - Pre‑fire vegetation within the SWC_118 prescribed burn cell at Yalgorup National Park, Western Australia.

Figure S4 – Fire moving through the SWC_118 prescribed burn cell at Yalgorup National Park, Western Australia.

Figure S5 – Fire moving through the SWC_118 prescribed burn cell at Yalgorup National Park, Western Australia.

Figure S6 – Post‑fire habitat conditions within the SWC_118 prescribed burn cell at Yalgorup National Park, Western Australia

Figure S7 – Area most impacted by the SWC_118 prescribed burn, due to high density of grass trees, at Yalgorup National Park, Western Australia.

Figure S8 – Refuge tree used by possum 150.022 during the passage of fire within the SWC_118 prescribed burn cell at Yalgorup National Park, Western Australia.

Figure S9 – Refuge tree used by possum 150.0362 during the passage of fire within the SWC_118 prescribed burn cell at Yalgorup National Park, Western Australia.

Figure S10 – Refuge tree used by possum 150.136 during the passage of fire within the SWC_118 prescribed burn cell at Yalgorup National Park, Western Australia.

Figure S11 – Refuge tree used by possum 150.036 during the passage of fire within the SWC_118 prescribed burn cell at Yalgorup National Park, Western Australia.

**Figure S12 -** Fine-scale activity (VeDBA, g) of all nine western ringtail possums (*Pseudocheirus occidentalis*) on their day of fire exposure, including the unexposed control (150.084). Red dashed lines indicate the time of ignition (10:00) at each possum's refuge location on its own fire day; grey shading indicates night.

**Figure S13 -** Residual diagnostics for the linear mixed models and the step-length model. Left: standardised residuals versus fitted values, with the red dashed line at zero. Right: normal Q-Q plots, with the red line showing the expected normal quantiles. Rows show the Table 3 diurnal and nocturnal models, the Table 4 diurnal and nocturnal models, and the Table 5 step-length model; the activity models were fitted to log-transformed VeDBA.
